# Supplementary material for: A previous hemorrhagic stroke protects against a subsequent stroke via microglia alternative polarization
Source: Commun Biol. 2022 Jul 2;5:654. doi: 10.1038/s42003-022-03621-4 (PMC9250506; doi:10.1038/s42003-022-03621-4)
Supplement: Supplementary file 2 — Supplementary Information [file 42003_2022_3621_MOESM2_ESM.pdf]

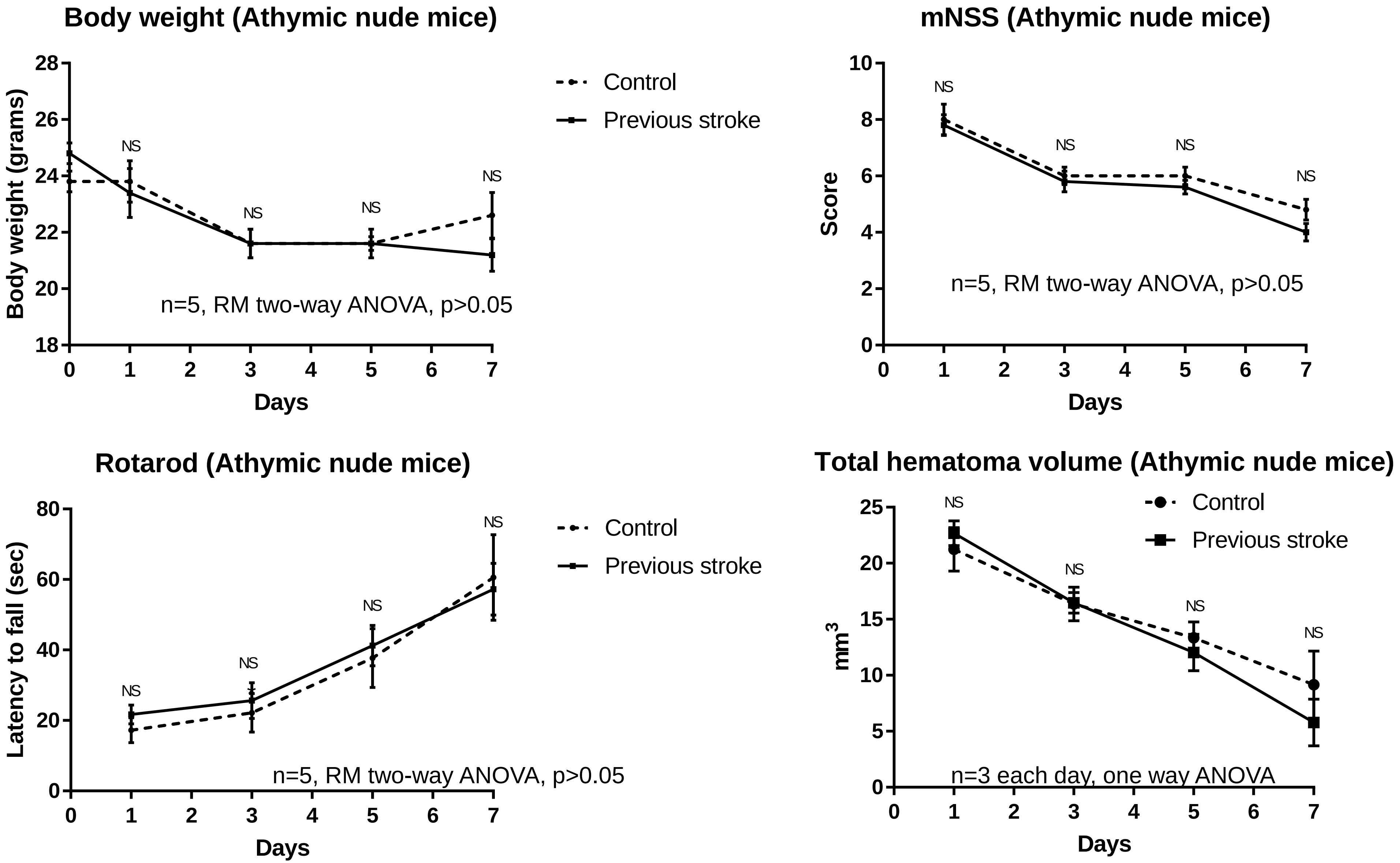


**Supplementary Figure 1.** Phenotypic and histological observations in athymic nude mice. The control group and the previous stroke group did not show significant difference in body weight, mNSS and Rotarod performance. Brain sections did not reveal difference in the total hematoma volumes.

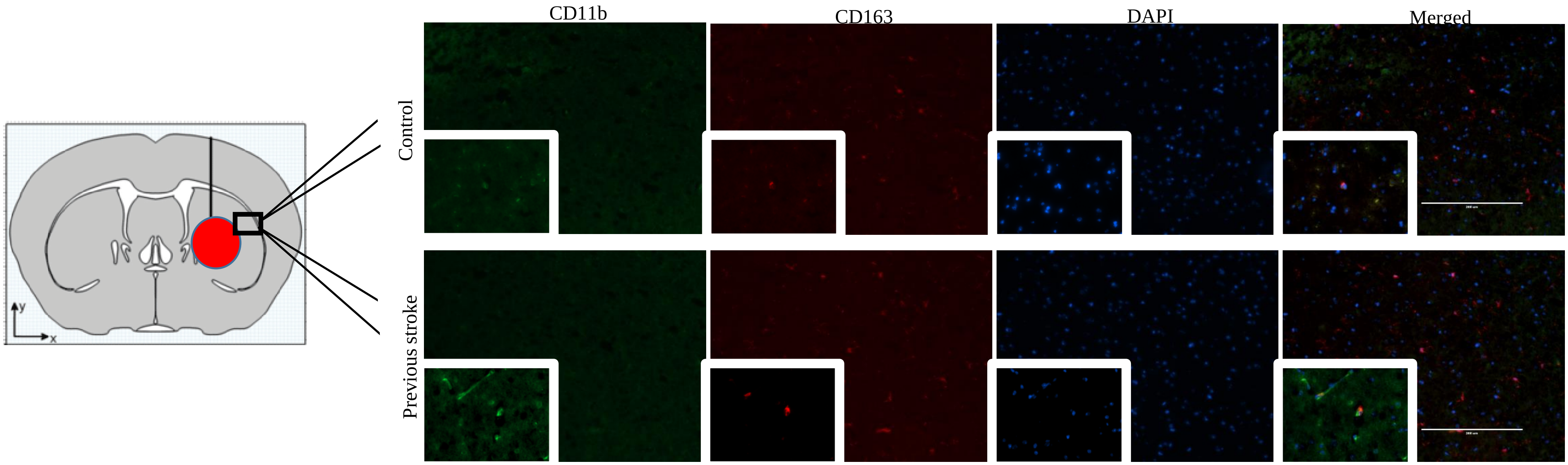


**Supplementary Figure 2.** Immunofluorescent staining of the perihematomal tissue of the control and previous stroke group.

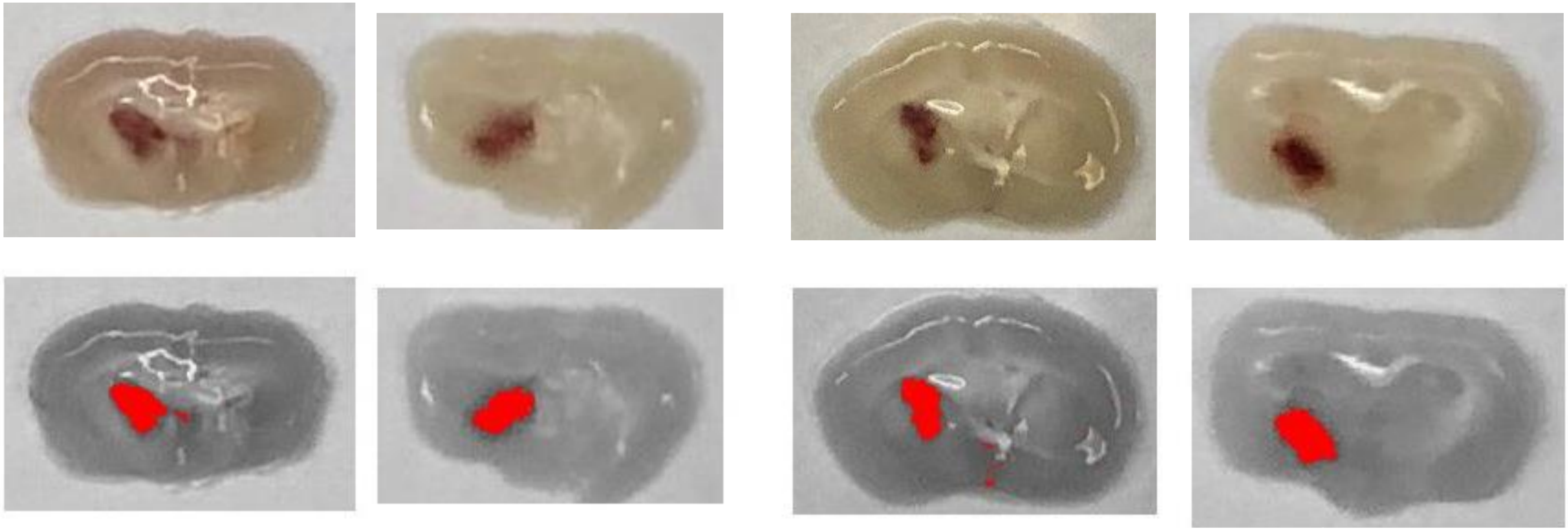

**1.98** **2.05** **2.04** **2.35 ($mm^2$)**

**Supplementary Figure 3**. Brain sections with the maximal hematoma area (expressed in $mm^2$) one day after the mini-stroke. Calculated areas demonstrate minimal inter-individual variation.
