# Supplementary material for: A previous hemorrhagic stroke protects against a subsequent stroke via microglia alternative polarization
Source: Commun Biol. 2022 Jul 2;5:654. doi: 10.1038/s42003-022-03621-4 (PMC9250506; doi:10.1038/s42003-022-03621-4)
Supplement: Supplementary file 5 — Reporting Summary [file 42003_2022_3621_MOESM5_ESM.pdf]

nature portfolio

Corresponding author(s): Gilberto Ka Kit Leung

Last updated by author(s): May 22, 2022

Reporting Summary

Nature Portfolio wishes to improve the reproducibility of the work that we publish. This form provides structure for consistency and transparency in reporting. For further information on Nature Portfolio policies, see our Editorial Policies and the Editorial Policy Checklist.

Statistics

For all statistical analyses, confirm that the following items are present in the figure legend, table legend, main text, or Methods section.

n/a	Confirmed	
☐	☒	The exact sample size (*n*) for each experimental group/condition, given as a discrete number and unit of measurement
☐	☒	A statement on whether measurements were taken from distinct samples or whether the same sample was measured repeatedly
☐	☒	The statistical test(s) used AND whether they are one- or two-sided<br>*Only common tests should be described solely by name; describe more complex techniques in the Methods section.*
☐	☒	A description of all covariates tested
☒	☐	A description of any assumptions or corrections, such as tests of normality and adjustment for multiple comparisons
☐	☒	A full description of the statistical parameters including central tendency (e.g. means) or other basic estimates (e.g. regression coefficient) AND variation (e.g. standard deviation) or associated estimates of uncertainty (e.g. confidence intervals)
☐	☒	For null hypothesis testing, the test statistic (e.g. *F*, *t*, *r*) with confidence intervals, effect sizes, degrees of freedom and *P* value noted<br>*Give P values as exact values whenever suitable.*
☐	☒	For Bayesian analysis, information on the choice of priors and Markov chain Monte Carlo settings
☒	☐	For hierarchical and complex designs, identification of the appropriate level for tests and full reporting of outcomes
☒	☐	Estimates of effect sizes (e.g. Cohen's *d*, Pearson's *r*), indicating how they were calculated

Our web collection on statistics for biologists contains articles on many of the points above.

Software and code

Policy information about availability of computer code

Data collection	N/A
Data analysis	GraphPad Prism version 6.04 for Windows, GraphPad Software

For manuscripts utilizing custom algorithms or software that are central to the research but not yet described in published literature, software must be made available to editors and reviewers. We strongly encourage code deposition in a community repository (e.g. GitHub). See the Nature Portfolio guidelines for submitting code & software for further information.

Data

Policy information about availability of data

All manuscripts must include a data availability statement. This statement should provide the following information, where applicable:

- Accession codes, unique identifiers, or web links for publicly available datasets
- A description of any restrictions on data availability
- For clinical datasets or third party data, please ensure that the statement adheres to our policy

The data of this study are available from the corresponding author on reasonable request.

Field-specific reporting

Please select the one below that is the best fit for your research. If you are not sure, read the appropriate sections before making your selection.

☒ Life sciences ☐ Behavioural & social sciences ☐ Ecological, evolutionary & environmental sciences

For a reference copy of the document with all sections, see nature.com/documents/nr-reporting-summary-flat.pdf

Life sciences study design

All studies must disclose on these points even when the disclosure is negative.

Sample size	As our experiment is a novel phenomenon, no estimated effect size could be calculated.
Data exclusions	Mice were excluded if a) death occurred within 1 hour of induction of anesthesia or b) no recovery from anesthesia within 10 hours.
Replication	Different subsets of mice underwent the same procedures.
Randomization	8 week old mice were ordered from the lab animal service center of our institution. Mice were randomly divided into groups prior to any procedure.
Blinding	Documentation of the animal behavioral experiments was conducted by a blinded observer. Calculation of MRI imaging results and hematoma volume was conducted by a blinded observer.

Reporting for specific materials, systems and methods

We require information from authors about some types of materials, experimental systems and methods used in many studies. Here, indicate whether each material, system or method listed is relevant to your study. If you are not sure if a list item applies to your research, read the appropriate section before selecting a response.

Materials & experimental systems

n/a	Involved in the study	
☐	☒	Antibodies
☒	☐	Eukaryotic cell lines
☒	☐	Palaeontology and archaeology
☐	☒	Animals and other organisms
☒	☐	Human research participants
☒	☐	Clinical data
☒	☐	Dual use research of concern

Methods

n/a	Involved in the study	
☒	☐	ChIP-seq
☐	☒	Flow cytometry
☐	☒	MRI-based neuroimaging

Antibodies

Antibodies used	Microglia surface marker CD11b (1:100 Cell Signaling, Danvers, MA, USA); M2 marker CD163 (1:120 Cell Signaling); CD11b (1:100 Cell Signaling, Danvers, MA, USA); secondary antibodies: anti-rabbit (Cell Signaling) and anti-mouse (Cell Signaling); DAPI nuclear staining (Sigma Aldrich). APC/Cy7 anti-mouse CD16/32 antibody, Biolegend #101327, 1:100; FITC anti-mouse CD206 antibody, Biolegend #141703, 1:80; Alexa Fluor 700 anti-mouse CD45.2 antibody, Biolegend #109822, 1:70; APC antimouse/human CD11b, Biolegend #101212, 1:100)
Validation	Validations and optimal concentrations of all the antibodies could be found on the manufacturer's websites (listed above).

Animals and other organisms

Policy information about studies involving animals; ARRIVE guidelines recommended for reporting animal research

Laboratory animals	C57BL/6N mice, Charles River Lab, USA
Wild animals	The study did not involve wild animals.
Field-collected samples	The study did not involve field-collected samples.
Ethics oversight	Committee on the Use of Live Animals in Teaching and Research, the University of Hong Kong

Note that full information on the approval of the study protocol must also be provided in the manuscript.

March 2021

Flow Cytometry

Plots

Confirm that:

☒ The axis labels state the marker and fluorochrome used (e.g. CD4-FITC).

☒ The axis scales are clearly visible. Include numbers along axes only for bottom left plot of group (a 'group' is an analysis of identical markers).

☒ All plots are contour plots with outliers or pseudocolor plots.

☒ A numerical value for number of cells or percentage (with statistics) is provided.

Methodology

Sample preparation	Peri-hematomal brain tissue was collected and mechanically homogenized by cutting with scissors, then digested by 5mL 4% collagenase (Sigma-Aldrich, St. Louis, MI, United States) for 15 minutes at 37°C. Digestion was halted by adding 5mL culture medium and single-cell suspension obtained by filtering through a 75µm cell strainer. Cells were centrifuged at 400g for 5 mins and resuspended in PBS. Cells were then washed again and resuspended in Flow Cytometry buffer (Cell signaling, Danvers, MA, United States) at a concentration of 100,000 cells per mL.<br>For in vitro studies, microglia were scraped from the flask by a cell scraper at the experimental endpoint of each group. Cells were washed twice by centrifuging at 400g for 5 minutes and suspended in flow cytometry buffer. Each mL of suspended cells was incubated with conjugated flow antibodies (APC/Cy7 anti-mouse CD16/32 antibody, Biolegend #101327, 1:100; FITC anti-mouse CD206 antibody, Biolegend #141703, 1:80; Alexa Fluor 700 anti-mouse CD45.2 antibody, Biolegend #109822, 1:70; APC antimouse/human CD11b, Biolegend #101212, 1:100) for 1 hour in darkness. Cells were then washed twice by centrifuging at 400g for 5 mins and resuspended in Flow Cytometry buffer.
Instrument	CytoFLEX Flow Cytometer (Beckman Life Sciences, Indianapolis, Indiana, United States)
Software	FlowJo™ v10.8.1
Cell population abundance	Whole brain tissues were lysed and analyzed and titrated to a concentration of 100,000 cells/mL. All cells were analyzed. For in vitro experiments, 10,000-20,000 cells were used per analysis.
Gating strategy	The same polygonal gating was used to determine leukocyte population within the FSC/SCC gating plot. CD45+/CD11b+ cells were gated by another polygon for all samples.

☒ Tick this box to confirm that a figure exemplifying the gating strategy is provided in the Supplementary Information.

Magnetic resonance imaging

Experimental design

Design type	T2 structural MRI on anesthetized mice to evaluate total lesion volume (including initial hematoma, surrounding edema, and peripheral hyperdense rim.)
Design specifications	Scout images were first acquired to determine the coronal and sagittal planes of the brain. Then, 12 coronal slices with 0.5/0.0mm thickness/gap were positioned.
Behavioral performance measures	Not applicable.

Acquisition

Imaging type(s)	Structural
Field strength	7T
Sequence & imaging parameters	T2 weighted images were acquired as anatomical reference using a Rapid Acquisition with Refocused Echoes (RARE) sequence (FOV=32×32 mm2,data matrix=256×256, RARE factor=8, TE/TR=36/4200ms).
Area of acquisition	Whole brain scan

Diffusion MRI ☐ Used ☒ Not used

Preprocessing

Preprocessing software	Images of the coronal plain would be imported into ImageJ for further analysis.
Normalization	For each slice of the 12 coronal images, the maximal injury area was outlined manually and area was converted from pixels to mm2 at a ratio of 1: 0.078125 *2 (Conversion ratio provided by manufacturer.) The sum of the combined injury area from the

	12 coronal slices were multiplied by slice thickness (0.5mm) to obtain the total volume of injured tissue (expressed in mm3).
Normalization template	Pixels to mm2 at a ratio of 1: 0.078125 *2 (Conversion ratio provided by manufacturer.)
Noise and artifact removal	Mice were fitted in prone position on a stereotactic holder to restrict head movement. Vital signs were monitored throughout the whole scanning process. Tail arterial oxygen saturation was monitored via a real-time feedback oximeter (target >95%); spontaneous respiratory rate was monitored via a motion sensor pad (Target: 56-60/min).
Volume censoring	N/A

Statistical modeling & inference

Model type and settings	Comparison of univariate effect between two groups
Effect(s) tested	Two-way ANOVA of the previous stroke group and control group

Specify type of analysis: ☒ Whole brain ☐ ROI-based ☐ Both

Statistic type for inference (See Eklund et al. 2016)	Voxel-wise
Correction	Holm-Bonferroni correction

Models & analysis

n/a	Involved in the study
☒	☐ Functional and/or effective connectivity
☒	☐ Graph analysis
☒	☐ Multivariate modeling or predictive analysis
